# 癌症患者维生素D营养状况分析

**DOI:** 10.3779/j.issn.1009-3419.2021.101.10

**Published:** 2021-05-20

**Authors:** 芷君 李, 静 时, 增亮 王, 海生 陈, 玉国 刘

**Affiliations:** 250117 济南，山东省肿瘤防治研究院（山东省肿瘤医 院），山东第一医科大学（山东省医学科学院） Shandong Cancer Hospital and Institute, Shandong First Medical University and Shandong Academy of Medical Sciences, Jinan 250117, China

**Keywords:** 维生素D, 25(OH)D, 维生素D缺乏, 癌症, Vitamin D, 25-hydroxyvitamin D, Vitamin D deficiency, Cancer

## Abstract

**背景与目的:**

维生素营养状况与癌症患者之间存在一定的关联，已有研究表明缺乏维生素会增加罹患癌症的风险，本研究的目的是了解癌症患者的维生素D营养状况，为进一步实施营养干预提供科学依据。

**方法:**

本研究回顾性分析了2017年7月-2019年6月在山东省肿瘤医院就诊的癌症患者血清25-羟维生素D的水平，采用SPSS 20.0统计软件进行单因素分析和多元线性回归分析。

**结果:**

本研究共纳入2, 487例癌症患者，平均血清25-羟维生素D的浓度为（12.70±6.82）ng/mL，纳入的癌症患者中维生素D缺乏者[25（OH）D < 20.00 ng/mL]占比92.20%。单因素分析显示：年龄、身体质量指数（body mass index, BMI）和癌症类型（*P* < 0.05）与维生素D缺乏显著相关。进一步的多因素分析显示，BMI（β=0.71）、年龄（β=-0.56）、季节（与夏季相比，冬季β=-0.99；秋季β=-0.76）和癌症的种类（与其他类型的癌症相比，肺癌β=-1.17；食管-胃癌β=-1.45；结直肠癌β=-1.05）与25-羟维生素D水平显著相关（*P* < 0.05）。

**结论:**

癌症患者25-羟维生素D水平偏低，存在严重的维生素D缺乏状况，其可能与年龄、BMI、季节和癌症类型有关，这表明应该加强对癌症患者维生素D水平的监测。

## Abstract (en)

**Background and objective:**

There is a certain correlation between vitamin nutritional status and cancer patients. Studies have shown that vitamin deficiency increases the risk of cancer. The purpose of this study is to understand the vitamin D nutritional status of cancer patients and to provide scientific basis for further nutritional intervention.

**Methods:**

Cancer patients who visited Shandong Cancer Hospital from July 2017 to June 2019 were included in this retrospective study. Serum 25-hydroxyvitamin D [25(OH)D] concentrations were measured. Univariate analysis and multiple linear regression analysis were carried out using SPSS 20.0.

**Results:**

A total of 2, 487 cancer patients were evaluable for this analysis. Mean 25(OH)D concentration was (12.70±6.82) ng/mL; the prevalence of vitamin D deficiency [25(OH)D concentration less than 20.00 ng/mL] was of 92.20%. In univariate analysis, age, body mass index (BMI), season and types of cancer were associated with 25(OH)D concentrations. In the multivariate analysis, BMI (β=0.71), age (β=-0.56), season (β=-0.99 for winter; β=-0.76 for autumn *vs* summer) and types of cancer (β=-1.17 for lung cancer; β=-1.45 for esophageal-gastric cancer; β=-1.05 for colorectal cancer *vs* other types of cancer) were independently and significantly associated with 25(OH)D levels (*P* < 0.05).

**Conclusion:**

Vitamin D deficiency was highly prevalent among cancer patients. Age, BMI, season and types of cancer may be associated with 25(OH)D levels, which indicate that monitoring of vitamin D level for cancer survivor should be taken into account.

维生素D是一种类固醇激素，主要来源于皮肤暴露在阳光下的内源性合成。天然维生素D进入身体后，在肝脏内羟基化形成25-羟基维生素D [25（OH）D]，然后在肾脏中被激活为1, 25-二羟基维生素D[1, 25（OH）_2_D_3_]^[1]^。1, 25（OH）_2_D_3_与维生素D受体调节各种维生素D应答基因的表达，大约有2, 000个基因受1, 25（OH）_2_D_3_分子调控^[1-4]^。由于维生素D调控较为广泛的靶标基因，不难理解维生素D在各种病理过程中发挥着重要作用，除调节钙、磷体内的代谢平衡和骨代谢外，还与自身免疫性疾病、代谢综合征、心血管疾病，甚至与癌症发展和进展有关^[1]^。

国内外实验和流行病学研究均已证明维生素D对癌症预防的重要性及维生素D缺乏与人类多种癌症相关^[5, 6]^，维生素D水平越高，癌症（如乳腺癌、结肠癌、肝癌）的发病率和死亡率越低^[5, 6]^。另外，由于癌症患者身体机能降低、饮食摄入量不足、化疗引起的相关症状以及较少外出暴露阳光等因素，更易出现维生素D缺乏的情况^[6]^。因此，近二十年来国内外筛查维生素D缺乏情况急剧增加。其他国家的一些研究^[7]^显示，在癌症幸存者中维生素D缺乏率占70%以上。然而，关于中国癌症患者维生素D水平的研究还未见报道。

维生素D在体内以25（OH）D作为主要储存形式，25（OH）D含量是国际上通行的衡量人体维生素D营养状况的指标，因此在本研究中我们回顾性分析了山东省肿瘤医院癌症患者血清25（OH）D浓度来评估维生素D的营养状况，并分析与其相关的影响因素，为进一步实施营养干预提供科学依据。

## 1 对象与方法

### 1.1 对象

回顾性分析2017年7月-2019年6月在山东省肿瘤医院就诊并检测血清25（OH）D水平的癌症患者，本研究方案经山东省肿瘤医院伦理委员会批准（伦理审批号：E2315）。

### 1.2 方法

#### 1.2.1 检测方法

25（OH）D检测由山东省肿瘤医院治疗药物检测室使用LK3000V维生素检测仪（天津兰标电子科技发展有限公司）测定^[8]^。简要步骤如下：①血样处理：首先采集肘静脉血1 mL，置于不含添加剂的普通血清真空采血管中，室温下4, 000 r/min离心8 min。然后取80 μL血清与2 mL样品处理溶液（天津兰标电子科技发展有限公司提供）混合；②电极活化：预处理：实验前，用镜头纸沾少许酒精，擦拭电极表面；实验中，如发现曲线不平滑或电极附着力差，则在绒布台抛光座上撒少许抛光粉，用超纯水浇湿，手握电极使垂直台座，以“8”字形打磨抛光；电极抛光后用超纯水冲洗，冲掉电极探头表面吸附的抛光粉及杂物。电极活化：将抛光好的工作电极分别插入对应电极插座，两根氯化银辅助电极针分别插在两边，使辅助电极紧贴工作电极，但两辅助电极不能互相接触，以免短路。将盛有2 mL样品处理液的样品管置于工作台旋转杯内，进行电极活化，检测项目电极活化均可在2 min之内完成；③样品检测：最后采用LK3000V维生素检测仪将混匀的样本处理液放置在工作台的旋转杯中，点击选择键进行检测。

#### 1.2.2 纳入与排除标准

纳入在山东省肿瘤医院就诊并进行血清25（OH）D检测的癌症患者，有多次入院记录的患者按照患者入院次数进行纳入统计。

#### 1.2.3 一般资料收集

通过病案查询获得调查对象的年龄、性别、身高、体重、25（OH）D浓度、检测25（OH）D的季节、所患癌症种类、从癌症诊断至检测25（OH）D的时间（简称诊断时长），并计算体重指数[身体质量指数（body mass index, BMI）=体重（kg）/身高的平方（m^2^）]。

#### 1.2.4 分类标准

维生素D营养状态划分标准：本研究根据美国内分泌协会临床实践指南^[9]^，维生素D缺乏定义为25（OH）D浓度低于20.00 ng/mL。

其他指标分类标准：年龄变量分为4组（≤17岁、18岁-40岁、41岁-65岁、≥66岁）。测试季节定义为：春季（3月-5月）、夏季（6月-8月）、秋季（9月-11月）和冬季（12月-2月）。BMI分为3组：体重不足（≤18.49 kg/m^2^）、正常（18.50 kg/m^2^-23.99 kg/m^2^）和肥胖（≥24.00 kg/m^2^）。诊断时长分为4组：0个月-3个月、4个月-12个月、13个月-36个月、≥37个月。所患癌种按部位分为乳腺癌、肺癌、食管-胃癌、结直肠癌和其他癌种。

### 1.3 统计学分析

采用SPSS 20.0统计软件进行数据整理和统计分析。计量资料以均数±标准差（Mean±SD）表示，计数资料以频率和百分比（%）表示。正态分布两独立样本比较采用独立样本*t*检验，非正态分布两独立样本比较选用非参数检验，计数资料比较采用卡方检验或*Fisher*精确检验。采用多元逐步回归法对影响25（OH）D浓度的因素进行分析。*P* < 0.05为差异有统计学意义。

## 2 结果

### 2.1 人群基本资料

该研究最终纳入2, 487例，其中男性1, 173例（47.17%），女性1, 314例（52.83%）。平均年龄为（55.20±12.80）岁（范围：5岁-90岁），癌症诊断包括乳腺癌（24.21%）、肺癌（13.67%）、食管-胃癌（16.89%）、结直肠癌（9.85%）及其他类型的癌症（35.38%）。25（OH）D的检测季节春、夏、秋、冬所占比例分别为37.39%、22.68%、18.82%和21.11%。诊断时长平均为（12.65±21.45）个月（范围：0个月-199个月）。25（OH）D浓度的平均值为（12.70±6.82）ng/mL。2, 293例患者25（OH）D水平低于20.00 ng/mL，25（OH）D缺乏比率为92.20%。

### 2.2 单因素分析结果

在单因素分析中，如表 1所示，年龄（*P*=0.02）、BMI（*P*=0.002）、季节（*P*=0.013）、癌症类型（*P* < 0.001）与25（OH）D浓度相关，这些因素纳入多元线性回归模型中。25（OH）D浓度在性别之间没有差异，与诊断时长也没有差异。表 2显示维生素D缺乏患病率在不同年龄段（*P*=0.049）、BMI（*P*=0.044）和癌症类型（*P*=0.003）中差异具有统计学意义。

**表 1:** 研究对象一般人口学特征及血清25（OH）D浓度水平（*n*=2, 487） Characteristics of study participants and comparison of 25(OH)D concentration according to clinical variables (*n*=2, 487)

Variables	*n*	%	Serum 25(OH)D concentration		*P*
			Mean±SD	SE	
Gender					0.220
Male	1, 173	47.17	12.87±7.17	0.21	
Female	1, 314	52.83	12.54±6.50	0.18	
Age (yr)					0.020
≤17	15	0.60	11.88±4.26	1.10	
18-40	287	12.14	13.53±8.23	0.49	
41-65	1, 638	65.86	12.75±6.58	0.16	
≥66	547	21.99	12.11±6.75	0.29	
BMI (kg/m^2^)					0.002
≤18.49	157	6.31	11.26±5.01	0.40	
18.50-23.99	1, 182	47.53	12.48±7.10	0.21	
≥24.00	1, 148	46.16	13.12±6.71	0.20	
Seasons					0.013
Spring	930	37.39	12.90±7.74	0.25	
Summer	564	22.68	13.27±6.46	0.27	
Autumn	468	18.82	12.31±6.24	0.29	
Winter	525	21.11	12.07±5.85	0.26	
Types of cancer					< 0.001
Lung cancer	340	13.67	12.13±6.12	0.33	
Breast cancer	602	24.21	12.85±5.59	0.23	
Esophageal-gastric cancer	420	16.89	11.71±5.69	0.28	
Colorectal cancer	245	9.85	12.35 ±5.77	0.37	
Other types of cancer	880	35.38	13.38±8.38	0.28	
Time since diagnosis (mon)					0.166
0-3	1, 261	50.70	12.97±7.53	0.21	
4-12	521	20.95	12.24±6.98	0.31	
13-36	438	17.61	12.42±5.04	0.24	
≥37	267	10.74	12.71±5.37	0.33	
25(OH)D: 25-hydroxy vitamin D; SD: standard deviations; BMI: body mass index. The 25(OH)D concentration was shown as mean and standard deviation (Mean±SD) in various variables. Categorical variables were reported as frequencies and percentages (%) and comparisons of 25(OH)D concentration in various variables were analyzed using One-way analysis of variance (*ANOVA*). The variables with* P* < 0.05 were selected in the multiple linear regression analysis. *P* < 0.05 indicates statistically significant differences of 25(OH)D concentration among various groups of categorical variables.					

**表 2:** 维生素D缺乏的分布情况 Distribution of participants'characteristics according to vitamin D deficiency

Variables	Serum 25(OH)D concentration		*P*
	< 20 ng/mL (*n*=2, 293)	≥20 ng/mL (*n*=194)	
Gender			0.940
Male	1, 081 (47.14%)	92 (47.42%)	
Female	1, 212 (52.86%)	102 (52.58%)	
Age (yr)			0.049
≤17	15 (0.65%)	0 (0.00%)	
18-40	256 (11.16%)	31 (15.98%)	
41-65	1, 507 (65.73%)	131 (67.53%)	
≥66	515 (22.46%)	32 (16.49%)	
BMI (kg/m^2^)			0.044
≤18.49	151 (6.59%)	6 (3.09%)	
18.50-23.99	1, 097 (47.84%)	85 (43.82%)	
≥24.00	1, 045 (45.57%)	103 (53.09%)	
Seasons			0.925
Spring	859 (37.46%)	71 (36.60%)	
Summer	517 (22.55%)	47 (24.23%)	
Autumn	434 (18.93%)	34 (17.52%)	
Winter	483 (21.06%)	42 (21.65%)	
Types of cancer			0.003
Lung cancer	321 (14.00%)	19 (9.79%)	
Breast cancer	559 (24.38%)	43 (22.16%)	
Esophageal-gastric cancer	397 (17.31%)	23 (11.86%)	
Colorectal cancer	229 (9.99%)	16 (8.25%)	
Other types of cancer	787 (34.32%)	93 (47.94%)	
Time since diagnosis (mon)			0.055
0-3	1, 146 (49.98%)	115 (59.28%)	
4-12	483 (21.06%)	38 (19.59%)	
13-36	415 (18.10%)	23 (11.86%)	
≥37	249 (10.86%)	18 (9.28%)	

### 2.3 多因素分析结果

在多因素分析中，多元线性回归模型确定了BMI（β=0.71, *P*=0.002）、年龄（β=-0.56, *P*=0.019）、季节（与夏季相比，冬季β=-0.99，*P*=0.004；秋季β=-0.76，*P*=0.036）和癌症的类型（与其他类型的癌症相比，肺癌β=-1.17，*P*=0.006；食管-胃癌β=-1.45，*P* < 0.001；结直肠癌β=-1.05，*P*=0.028）与25（OH）D浓度相关（表 3）。25（OH）D的浓度与BMI呈显著正相关，和年龄呈负相关。与夏季相比，25（OH）D浓度在冬季和秋季较低。与其他种类癌症患者相比，肺癌、食管-胃癌或结直肠癌患者的25（OH）D浓度较低。

**表 3:** 多元线性回归分析中25（OH）D浓度的决定因素 The determinants of 25(OH)D concentration in multiple linear regression analysis

Item	*β* (95%CI)	*t*	*P*	*β* (standardized coefficients)
Age (yr)	0.56 (-1.04-0.09)	-2.34	0.019	-0.049
BMI (kg/m^2^)	0.71 (0.26-1.16)	3.11	0.002	0.063
Season				
Autumn (Autumn *vs* summer)	-0.76 (-1.47-0.05)	-2.10	0.036	-0.043
Winter (Winter *vs* summer)	-0.99 (-1.67-0.32)	-2.88	0.004	-0.059
Types of cancer				
Lung cancer (Lung cancer *vs* other types of cancer)	-1.17 (-2.00-0.34)	-2.76	0.006	-0.059
Esophageal-gastric cancer (Esophageal-gastric cancer *vs* other types of cancer)	-1.45 (-2.25-0.64)	-3.52	< 0.001	-0.079
Colorectal cancer (Colorectal cancer *vs* other types of cancer)	-1.05 (-1.98-0.12)	-2.20	0.028	-0.046

## 3 讨论

在我们的研究中，癌症患者维生素D缺乏的患病率高达92.20%，高于文献报道的中国普通人群的数据（64.70%-89.00%）^[10-15]^。这一结果与国外的大多数研究一致。Tangpricha等^[16]^和Sinha等^[17]^报道了两项病例对照研究均显示癌症患者维生素D缺乏的比例高于健康对照组，但另一项韩国的病例对照研究^[18]^显示癌症幸存者中维生素D缺乏的患病率与对照组没有明显差异（62.70% *vs* 62.70%）。这些研究不一致的结果可能是由于研究设计、样本大小、研究人口、癌症诊断、癌症治疗相关的因素及维生素D的补充和评估方法造成的。与之前的大多数研究^[19-22]^一致，我们观察到老年人维生素D缺乏的风险更高。这是因为衰老降低了人体皮肤产生足够数量维生素D的能力，减少了从食物中吸收维生素D的能力。我们的研究^[12, 22]^还发现，冬季和秋季的25（OH）D检测浓度低于夏季。之前的报告也显示了类似的结果，这可能是因为人们在寒冷的季节缺乏足够的阳光照射和花更少的时间在户外运动上。

有研究^[22-25]^提出，肥胖人群中25（OH）D浓度较低是由于多种因素造成的，如脂肪中维生素D的过度隔离和储存、体内脂肪池的清除增加以及日照减少。然而，在本研究中，我们发现维生素D的水平随着BMI的增加而持续下降，这可能是由于我们的研究中体重不足的受试者比例很小（6.30%）。此外，还有研究^[26]^并不认为BMI是肥胖的可靠指标，这是因为它不能区分脂肪组织和其他组织。

在我们的研究中，肺癌、食管-胃癌、结直肠癌患者维生素D缺乏的风险高于其他类型的癌症患者，乳腺癌患者与其他癌症患者相比维生素D缺乏无统计学差异。维生素D缺乏已发现与多种癌症^[3, 7, 27]^有关，包括结直肠癌、前列腺癌、肺癌和乳腺癌等。然而，大多数研究都局限于样本相对较小、某一种特定类型的癌症，对不同类型癌症幸存者维生素D缺乏情况的比较目前还缺乏流行病学证据。

我们还查询了关于癌症患者如何补充维生素D的相关文献^[6, 28]^。这些研究提出对维生素D不足患者推荐的剂量范围为8, 000 IU-50, 000 IU，每周1次-3次^[6]^。然而，另一篇关于癌症患者补充维生素D的综述^[28]^显示，维生素D补充剂提高循环骨化二醇水平的功效尚不清楚，标准的补充方案维生素D3 < 1, 000 IU/d可能不能维持足够的水平。这些也提示我们在进行抗癌治疗的同时，还应考虑对癌症患者维生素D水平的监测和维生素D的补充。

根据我们查阅的文献，目前还未见关于中国癌症患者维生素D营养状况的报道，本文尚属首次。然而，我们的研究仍然存在一些局限性。由于研究的设计，一些重要的变量，如饮食习惯、阳光照射和维生素D的补充没有在所有的受试者中获得，化疗、放疗、手术等癌症治疗因素也未纳入分析。

综上所述，癌症患者维生素D缺乏程度较高。年龄、BMI、季节、所患癌种均可能影响维生素D的水平。这提示我们应该考虑加强对癌症患者维生素D水平的监测及适当补充，但还需要进一步的临床研究并评估维生素D水平与癌症患者生存结果之间的因果关系。

## References

[1] Adams JS, Hewison M (2010). Update in vitamin D. J Clin Endocrinol Metab.

[2] Cai GH, Li MX, Lu L (2015). The current role and therapeutic targets of vitamin D in gastrointestinal inflammation and cancer. Curr Pharm Des.

[3] Nabi G, Hobani Y, Sarwat M (2015). High prevalence of vitamin D deficiency and cancer in Saudi Arabian populations: Can we hypothesize a link?. Med Hypotheses.

[4] Nagpal S, Na S, Rathnachalam R (2005). Noncalcemic actions of vitamin D receptor ligands. Endocr Rev.

[5] Mondul AM, Weinstein SJ, Layne TM (2017). Vitamin D and cancer risk and mortality: state of the science, gaps, and challenges. Epidemiol Rev.

[6] Garland CF, Gorham ED, Mohr SB (2009). Vitamin D for cancer prevention: global perspective. Ann Epidemiol.

[7] Gupta D, Vashi PG, Trukova K (2011). Prevalence of serum vitamin D deficiency and insufficiency in cancer: Review of the epidemiological literature. Exp Ther Med.

[8] Wang ND, Guo X, Chen KG (2015). Clinical application of vitamin for therapy and diagnostic monitoring. Zhongguo Yi Yuan Yao Xue Za Zhi.

[9] Holick MF, Binkley NC, Bischoff-Ferrari HA (2011). Evaluation, treatment, and prevention of vitamin D deficiency: an Endocrine Society clinical practice guideline. J Clin Endocrinol Metab.

[10] Gao Q, Liu Y (2012). Research progress of vitamin D deficiency in Chinese population. Zhongguo Gong Gong Wei Sheng.

[11] Zhou JL, Chen WJ, Wu GC (2009). Research progress of vitamin D supplement. Zhonghua Er Ke Za Zhi.

[12] Wang S, Shen G, Jiang S (2017). Nutrient status of vitamin D among Chinese children. Nutrients.

[13] Zhang RP, Yu SL, Cheng XQ (2017). Survey of vitamin D status in apparently healthy younger and elder adults. Zhonghua Jian Yan Yi Xue Za Zhi.

[14] Fang HL, Yu SL, Han JH (2014). Serum 25-hydroxy vitamin D3 and 25-hydroxy vitamin D2 levels in healthy population from North China. Zhonghua Gu Zhi Shu Song He Gu Kuang Yan Ji Bing Za Zhi.

[15] Gu CL (2016). Surveillance and analysis of children's 25-hydroxyvitamin D levels in different regions of my country. Zhongguo You Sheng Yu Yi Chuan Za Zhi.

[16] Tangpricha V, Colon NA, Kaul H (2004). Prevalence of vitamin D deficiency in patients attending an outpatient cancer care clinic in Boston. Endocr Pract.

[17] Sinha A, Avery P, Turner S (2011). Vitamin D status in paediatric patients with cancer. Pediatr Blood Cancer.

[18] Oh MG, Han MA, Park J (2015). The prevalence of vitamin D deficiency among cancer survivors in a nationwide survey of the Korean population. PLoS One.

[19] MacLaughlin J, Holick MF (1985). Aging decreases the capacity of human skin to produce vitamin D3. J Clin Invest.

[20] Ebeling PR, Sandgren ME, DiMagno EP (1992). Evidence of an age-related decrease in intestinal responsiveness to vitamin D: relationship between serum 1, 25-dihydroxyvitamin D3 and intestinal vitamin D receptor concentrations in normal women. J Clin Endocrinol Metab.

[21] van der Wielen RP, Lowik MR, van den Berg H (1995). Serum vitamin D concentrations among elderly people in Europe. Lancet.

[22] Lagunova Z, Porojnicu AC, Lindberg F (2009). The dependency of vitamin D status on body mass index, gender, age and season. Anticancer Res.

[23] Han SS, Kim M, Lee SM (2014). Association between body fat and vitamin D status in Korean adults. Asia Pac J Clin Nutr.

[24] Arunabh S, Pollack S, Yeh J (2003). Body fat content and 25-hydroxyvitamin D levels in healthy women. J Clin Endocrinol Metab.

[25] Lagunova Z, Porojnicu AC, Grant WB (2010). Obesity and increased risk of cancer: does decrease of serum 25-hydroxyvitamin D level with increasing body mass index explain some of the association?. Mol Nutr Food Res.

[26] VanItallie TB, Yang MU, Heymsfield SB (1990). Height-normalized indices of the body's fat-free mass and fat mass: potentially useful indicators of nutritional status. Am J Clin Nutr.

[27] Budhathoki S, Hidaka A, Yamaji T (2018). Plasma 25-hydroxyvitamin D concentration and subsequent risk of total and site specific cancers in Japanese population: large case-cohort study within Japan Public Health Center-based Prospective Study cohort. BMJ.

[28] Teleni L, Baker J, Koczwara B (2013). Clinical outcomes of vitamin D deficiency and supplementation in cancer patients. Nutr Rev.

